# Levels of anxiety in women aged ≥45 years undergoing diagnostic large loop excision of the transformation zone: A longitudinal study

**DOI:** 10.1111/1471-0528.17299

**Published:** 2022-10-13

**Authors:** Line Winther Gustafson, Mette Bach Larsen, Anne Hammer, Lone Kjeld Petersen, Berit Andersen, Pinar Bor

**Affiliations:** ^1^ Department of Public Health Programmes University Research Clinic for Cancer Screening, Randers Regional Hospital Randers Denmark; ^2^ Department of Clinical Medicine Aarhus University Aarhus Denmark; ^3^ Department of Obstetrics and Gynaecology Aarhus University Hospital Aarhus Denmark; ^4^ Department of Obstetrics and Gynaecology NIDO – Centre for Research and Education, Gødstrup Hospital Herning Denmark; ^5^ Department of Obstetrics and Gynaecology Odense University Hospital and Open Patient Data Explorative Network Odense Denmark; ^6^ Department of Clinical Research University of Southern Denmark Odense Denmark; ^7^ Department of Obstetrics and Gynaecology Randers Regional Hospital Randers Denmark

**Keywords:** anxiety, cervical cancer screening, colposcopy, LLETZ, postmenopausal women, STAI

## Abstract

**Objective:**

To measure anxiety levels in women aged ≥45 years undergoing diagnostic large loop excision of the transformation zone (LLETZ) at the first colposcopy visit.

**Design:**

Longitudinal study.

**Setting:**

Three colposcopy clinics in the Central Denmark Region.

**Population:**

Women aged ≥45 years undergoing diagnostic LLETZ.

**Methods:**

Women completed the State–Trait Anxiety Inventory (STAI) and Short Form 12 (mental and physical health) questionnaires before, immediately after, and at 1 and 6 months after LLETZ.

**Main outcome measures:**

STAI state anxiety median scores were calculated and stratified by health status, by letter with information about screening result and by LLETZ results.

**Results:**

Of 109 eligible women, 11 were excluded, leaving 98 women for the final analyses. Response rates ranged from 84.7% to 100%. Overall, state anxiety levels were low; however, a decrease was observed from before to immediately after the LLETZ (33.4 vs 29.3, *p* < 0.001). The anxiety levels remained stable up to 6 months after LLETZ. Women with poor mental health were more likely to have higher anxiety levels compared with women with good mental health (before LLETZ, RR 3.77, 95% CI 2.12–6.70; 1 month after LLETZ, RR 3.37, 95% CI 1.59–7.15; 6 months after LLETZ, RR 1.93, 95%CI 1.06–3.51).

**Conclusions:**

Overall, colposcopy and diagnostic LLETZ in women aged ≥45 years were not associated with high levels of anxiety. Anxiety levels were highest before colposcopy, and the women seemed to experience immediate relief afterwards. Women with poor mental health had the highest anxiety levels throughout the study, which might call for special attention.

## Abstract 2

**Linked article**: This article is commented on by Margaret Cruickshank, pp. 201 in this issue. To view this mini commentary visit https://doi.org/10.1111/1471-0528.17336.

## 1 INTRODUCTION

Cervical cancer screening programmes have dramatically decreased the incidence of and mortality from cervical cancer.^1^ However, women aged ≥60 years have a higher incidence of and mortality from cervical cancer than younger women^2^, ^3^ indicating that the screening or the diagnostic work‐up and subsequent treatment of these women may be suboptimal. The reasons for this are likely to be multifactorial,^4^ but may partly be explained by age‐dependent changes of the cervix in postmenopausal women, such as a retraction of the transformation zone (TZ) into the cervical canal as a result of decreasing estrogen levels.^5^ These changes challenge the performance of colposcopy and may lead to underdiagnosis, as the lesion may be located in the cervical canal.^6^ To avoid underdiagnosis in postmenopausal women, one strategy could be to offer diagnostic large loop excision of the transformation zone (LLETZ), even though this entails a risk of overtreatment. A recent qualitative study on women aged ≥60 years showed that women preferred a diagnostic LLETZ over clinical follow‐up, despite the fact that having a diagnostic LLETZ may result in an increased risk of overtreatment.^7^ It is well known that younger women have increased anxiety in relation to referral for and performance of colposcopy and in regards to their future fertility potential.^8^, ^9^, ^10^, ^11^, ^12^ However, the extent of anxiety in postmenopausal women when offering diagnostic LLETZ is unknown.

This study aimed to investigative changes in anxiety levels over time in women aged ≥45 years with abnormal screening test results undergoing diagnostic LLETZ at the first colposcopy visit. Furthermore, we wanted to explore whether the anxiety level differed according to the woman’s physical and/or mental health, previous screening history, information provided in the letter with the screening result and the final histological result of the LLETZ specimen.

## 2 METHODS

### 2.1 Setting

In Denmark, organised cervical cancer screening is offered to women aged 23–64 years.^13^ In addition, as part of a research project, women residing in the Central Denmark Region aged 65–69 years have been invited to an additional human papillomavirus (HPV) test since April 2019.^14^ Women with an abnormal cervical cancer screening test are either referred for colposcopy or undergo repeat testing, depending on the screening result (Table S1). Screening, diagnostic work‐up and any subsequent treatment are all free of charge.

### 2.2 Study design

The study was designed as a longitudinal study based on questionnaire data from a clinical study carried out at the Departments of Obstetrics and Gynaecology in the Central Denmark Region (i.e. Randers, Horsens and Viborg) from March 2019 through June 2021.^6^ Briefly, women aged ≥45 years referred for colposcopy as a result of an abnormal cervical cancer screening test had a colposcopic examination performed. Only women with a transformation zone type 3 (TZ3; defined according to 2011 International Federation of Cervical Pathology and Colposcopy nomenclature) were included.^5^ According to the study set‐up, multiple biopsies were taken and a diagnostic LLETZ was performed with local anaesthesia immediately after colposcopic examination.^6^ Project information and a survey link to Q1, the State–Trait Anxiety Inventory (STAI) and Short Form 12 (SF‐12), were attached to the appointment letter. All women referred were encouraged to complete the questionnaire prior to the colposcopy visit. Q2 (STAI) was completed immediately after colposcopy and LLETZ. Q3 and Q4 both included STAI and SF‐12, sent by secure digital mail 1 and 6 months after the LLETZ.

All women received oral and written information, and the participating women signed a consent form. Women who completed Q2 immediately after LLETZ were included. Women were excluded if Q1 was completed on the day of LLETZ, or after, and/or Q2 was completed after the day of LLETZ.

### 2.3 Data sources and definitions

In this study we used the Danish version of the STAI,^15^, ^16^ which evaluates current anxiety in relation to a specific event (e.g. colposcopy and LLETZ). The scale consists of 20 items using a 4‐point Likert scale.^15^ The score ranges from 20 to 80 points, with a higher score indicating increased levels of anxiety. According to the STAI manual, among working adult women in the USA the mean scores for state anxiety were 36.03 (SD 11.07) in the group aged 45–49 years and 32.20 (SD 8.67) in the group aged 50–69 years.^15^ Thus, in this study, women with a score of <35 were considered to have low anxiety levels and women with a score of ≥35 were considered to have increased anxiety levels.

Health‐related quality of life was measured using the SF‐12 questionnaire, which consists of 12 general health‐related quality of life questions. The range of SF‐12 is from 0 to 100, with a high score indicating a high quality of life.^17^ According to the SF‐12 manual, the physical and mental mean scores for females in the USA aged 65–74 years was 43.65 (SD 11.02) and 52.10 (SD 9.53), respectively.^18^ Good physical and mental health were defined as mean scores of ≥44 and ≥52, respectively.

Information on previous abnormal screening results, the results of the recent screening test and the histopathological result of the LLETZ specimen were obtained from the Danish Pathology Databank, which is a nationwide registry storing results on all cytological and histological specimens at an individual level since 1997.^19^ Information about the screening result was grouped according to the information that the women received in a standardised letter providing them with the result of the abnormal screening test and leading to the referral: either HPV positive or abnormal cytology, defined by atypical squamous cells of undetermined significance (ASC‐US) or worse (ASC‐US+) (according to the Bethesda 2014 grading system)^20^ with atypical squamous cells of undetermined significance (ASC‐US) or worse (ASC‐US+). The histological result of the LLETZ specimens was classified according to the cervical intraepithelial neoplasia (CIN) classification, and grouped as either <CIN2 (normal and CIN1) or CIN2+ (CIN2, CIN3, adenocarcinoma in situ, unclassifiable CIN and cancer).^21^

### 2.4 Data collection

In Denmark, approximately 90% of the citizens communicate with the Danish health authorities, general practitioner and the hospitals by secure digital mail.^22^ If the woman did not have secure digital mail, a paper questionnaire was sent by postal mail. A reminder letter was sent 2 weeks after they received the questionnaire, in the case of no response, and if women did not reply they were contacted by phone. After the LLETZ was performed, women completed a questionnaire on basic characteristic and behavioural factors (e.g. weight, smoking, alcohol and sexual behaviour).

Data from registries and electronic patient’s records were continuously collected during the study period and stored using Research Electronic Data Capture (REDCap).^23^, ^24^

No patients or patient organisations were involved in the development, design or implementation of this study.

### 2.5 Statistical analyses

Basic characteristics of the included women were reported as number and percentages, and continuous variables were reported as median values with corresponding interquartile ranges (IQRs). The median anxiety score with corresponding IQR was calculated at four different time points: before LLETZ, immediately after LLETZ, and 1 and 6 months after LLETZ.

A Wilcoxon matched‐pairs signed‐rank test was used to analyse differences in median anxiety scores over time. We calculated relative risks (RRs) of increased anxiety levels (i.e. STAI ≥ 35) with 95% confidence intervals (95% CIs) using generalised linear models for the binomial family. We conducted a sensitivity analysis using an STAI median score of ≥40 as the threshold for increased anxiety.

All results were reported overall and then stratified by physical and mental health estimated using SF‐12 at baseline (i.e. before LLETZ) (good vs poor), information about screening result (HPV positive vs abnormal cytology), previous abnormal cervical cytology result (normal vs abnormal) and histological result of the LLETZ (<CIN2 vs CIN2+).

All analyses were performed using STATA 17 (StataCorp LLC, College Station, TX, USA); *p* ≤ 0.05 was considered statistically significant.

## 3 RESULTS

### 3.1 Study population

**FIGURE 1:**
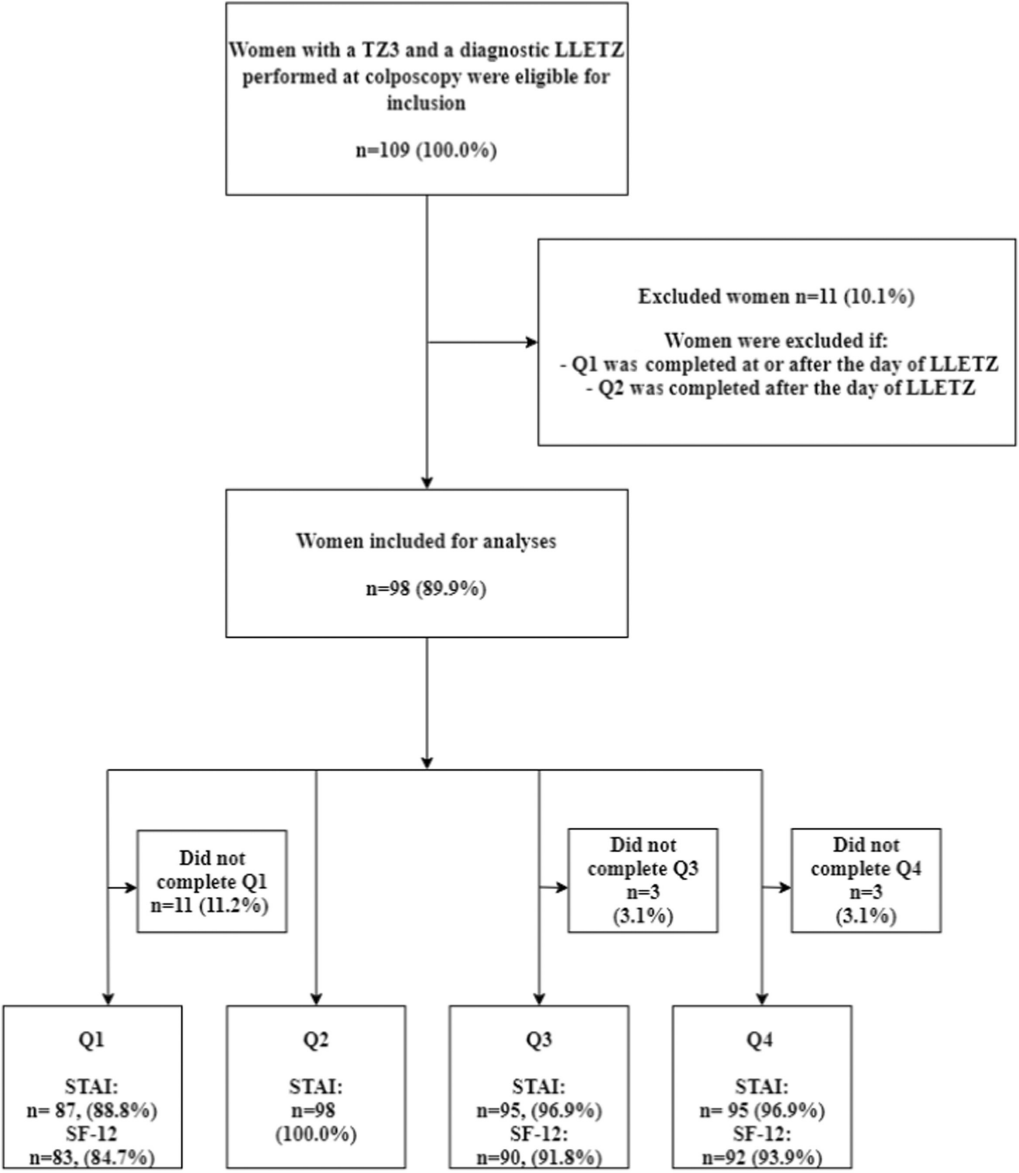
Flow chart of women undergoing LLETZ and completing Q1–Q4

A total of 109 women were eligible for inclusion as they had a TZ3 and a diagnostic LLETZ performed. Of these, 11 (6.5%) women were excluded as their questionnaires had not been completed in time, resulting in 98 (89.9%) women being included for the final analyses (Figure 1).

The median age of the included women was 67.7 years (IQR 61.6–70.3 years), and 91 (92.9%) women were postmenopausal. Most women were non‐smokers (72.5%) and their median body mass index (BMI) was 24.5 kg/m^2^ (IQR 21.8–28.2 kg/m^2^) (Table 1). Forty‐five women received a letter with the information that they were HPV positive (45.9%) and 53 (54.1%) women received a letter indicating that they had an abnormal cytology result (Table 1).

**TABLE 1:** Basic characteristics of women with TZ3 who underwent diagnostic LLETZ at their first colposcopy visit (*N* = 98)

Characteristics	*n* (%)
Median age (IQR), years	67.7 (61.6–70.3)
Age groups	
45–59 years	16 (16.3)
≥60 years	82 (83.7)
Menopausal status	
Premenopausal	2 (2.0)
Postmenopausal	91 (92.9)
Missing	5 (5.1)
Median BMI (IQR), kg/m^2^	24.5 (21.8–28.2)
Smoking	
Yes	23 (23.5)
No	71 (72.5)
Alcohol consumption^a^	
0–7	76 (77.6)
>7	19 (19.4)
Previous abnormal cervical cytology (ASC‐US+^b^)	30 (30.6)
Letter with information about the screening result^c^	
HPV positive	45 (45.9)
Abnormal cytology	53 (54.1)
Histology results^d^	
<CIN2	65 (66.3)
CIN2+	33 (33.7)

Abbreviations: IQR, interquartile range; LLETZ, large loop excision of the transformation zone.

^a^ A maximum of 7 units of alcohol is recommend by the Danish Health Authority.

^b^ ASC‐US+ is defined as atypical squamous cells of undetermined significance (ASC‐US), low‐grade squamous intraepithelial lesion (LSIL), atypical squamous cells that cannot exclude high‐grade squamous intraepithelial lesion (ASC‐H) and high‐grade squamous intraepithelial lesion (HSIL).

^c^ Information about the screening result was divided between a letter stating that they were HPV positive or a letter stating that they had abnormal cytology (ASC‐US+).

^d^ <CIN2, normal and CIN1; CIN2+, CIN2, CIN3, adenocarcinoma in situ and cancer.

All included women completed Q2 (100.0%), whereas the STAI in Q1, Q3 and Q4 was completed by 87 (88.8%), 95 (96.9%) and 95 (96.9%) women, respectively. The SF‐12 scale in Q1, Q3 and Q4 was completed by 83 (84.7%), 90 (91.8%) and 92 (93.9%) women, respectively (Figure 1).

### 3.2 Median STAI score over time

Overall, the anxiety levels decreased significantly from before the LLETZ to immediately after the LLETZ (32.0 vs 28.0, *p* < 0.001). The anxiety levels remained stable over time, from immediately after the LLETZ to 1 month after the LLETZ (28.0 vs 30.0, *p* = 0.155), and from 1 to 6 months after the LLETZ (30.0 vs 29.0, *p* = 0.278) (Table 2).

**TABLE 2:** Median anxiety score over time for women undergoing LLETZ, overall and stratified by good or poor health (SF‐12 – baseline), by previous abnormal cervical cytology, by letter with information about screening result and by histology result of the LLETZ

	Before LLETZ	Immediately after LLETZ	1 month after LLETZ	6 months after LLETZ
	Median, IQR			
Total	32.0 (23.0–40.0)	28.0 (23.0–35.0)	30.0 (23.0–35.0)	29.0 (23.0–39.0)
SF‐12 physical^a^				
Good physical health (≥44)	30.0 (23.0–36.0)	27.0 (21.0–35.0)	26.5 (22.0–34.0)	26.0 (22.0–32.5)
Poor physical health (<44)	36.5 (28.0–46.0)	28.0 (24.0–33.0)	32.0 (27.0–35.0)	37.5 (23.5–44.5)
SF‐12 mental^a^				
Good mental health (≥52)	27.0 (22.0–33.0)	27.0 (21.0–31.0)	27.0 (23.0–33.0)	25.0 (21.0–33.0)
Poor mental health (<52)	41.0 (34.0–48.0)	29.0 (24.0–37.0)	34.0 (23.0–42.0)	33.0 (26.0–44.0)
Previous abnormal cervical cytology (ASC‐US+)				
Normal cytology	30.0 (23.0–37.0)	27.0 (21.0–31.5)	28.0 (23.0–34.0)	28.5 (22.0–35.0)
Abnormal cytology	34.0 (25.0–42.0)	30.0 (26.0–37.0)	33.5 (23.0–42.0)	30.0 (25.0–41.0)
Letter with information about screening result^b^				
Positive HPV	30.0 (23.5–41.0)	28.0 (24.0–36.0)	30.0 (23.0–34.0)	27.0 (22.0–39.0)
Abnormal cytology	33.0 (23.0–39.0)	27.0 (23.0–33.0)	28.0 (23.0–37.0)	30.0 (23.5–39.0)
Histology results^c^				
<CIN2	30.0 (23.0–41.0)	27.0 (23.0–34.0)	27.0 (22.0–34.0)	27.0 (22.0–35.0)
CIN2+	33.0 (27.0–37.0)	30.0 (24.0–35.0)	33.5 (26.0–42.0)	31.5 (26.0–41.5)

Abbreviations: LLETZ, large loop excision of the transformation zone; IQR, interquartile range.

^a^ Short Form 12 (SF‐12).

^b^ Information about the screening result was divided between a letter stating that they were HPV positive or a letter stating that they had abnormal cytology (ASC‐US+).

^c^ <CIN2, normal and CIN1; CIN2+, CIN2, CIN3, adenocarcinoma in situ and cancer. ASC‐US+ is defined as atypical squamous cells of undetermined significance (ASC‐US), low‐grade squamous intraepithelial lesion (LSIL), atypical squamous cells that cannot exclude high‐grade squamous intraepithelial lesion (ASC‐H) and high‐grade squamous intraepithelial lesion (HSIL).

In women with good physical health (SF‐12) a slight but non‐significant decrease was observed from before to immediately after the LLETZ (30.0 vs 27.0, *p* = 0.093), after which the STAI score remained stable at 1 and 6 months after the LLETZ (27.0 vs 26.5, *p* = 0.848 and 26.5 vs 26.0, *p* = 0.701, respectively). The anxiety level in women with poor physical health (SF‐12) decreased significantly from before to immediately after the LLETZ (36.5 vs 28.0, *p* < 0.001), after which it remained stable at 1 and 6 months after the LLETZ (28.0 vs 32.0, *p* = 0.045 and 32.0 vs 37.5, *p* = 0.383, respectively) (Table 2). In women with poor mental health (SF‐12) the anxiety levels decreased significantly from before the LLETZ to immediately after the LLETZ (41.0 vs 29.0, *p* < 0.001), and remained below 40 at 1 and 6 months after the LLETZ (29.0 vs 34.0, *p* = 0.332 and 34.0 vs 33.0, *p* = 0.275, respectively) (Table 2). In women with good mental health (SF‐12) the anxiety levels were stable before, immediately after, and 1 and 6 months after the LLETZ (27.0 vs. 27.0, *p* = 0.618, 27.0 vs 27.0, *p* = 0.474, and 27.0 vs. 25.0, *p* = 0.844, respectively) (Table 2).

In women with previous abnormal cervical cytology (i.e. ASC‐US+) the anxiety level decreased significantly from 34.0 to 30.0 after the LLETZ (*p* = 0.035), and remained fairly stable at 1 and 6 months after the LLETZ (30.0 vs 33.5, *p* = 0.423 and 33.5 vs 30.0, *p* = 0.900, respectively) (Table 2).

With respect to the letter with information about the screening result, the anxiety level decreased significantly from before the LLETZ to immediately after te LLETZ in women referred with abnormal cytology and in women who were HPV positive (33.0 vs 27.0, *p* = 0.003 and 30.0 vs 28.0, *p* = 0.013, respectively) (Table 2). When stratifying by histology result (<CIN2 and CIN2+), the anxiety levels decreased significantly from before the LLETZ to immediately after the LLETZ for both groups (30.0 vs 27.0, *p* = 0.001 and 33.0 vs 30.0, *p* = 0.024, respectively). In women diagnosed with CIN2+, the anxiety level increased non‐significantly from immediately after the LLETZ to 1 month after the LLETZ (30.0 vs 33.5, *p* = 0.127), after which it remained stable (33.5 vs 31.5, *p* = 0.952) (Table 2).

### 3.3 Relative risk of increased anxiety level

Women with poor mental health were significantly more likely to have high anxiety levels immediately after the LLETZ, and at 1 and 6 months after the LLETZ, compared with women with good mental health (RR 2.07, 95% CI 1.03–4.17; RR 3.37, 95% CI 1.59–7.15; and RR 1.93, 95% CI 1.06–3.51, respectively) (Table 3). The same pattern was not seen when stratifying for physical health (RR 0.82, 95% CI 0.36–1.86; RR 1.64, 95% CI 0.79–3.41; and RR 2.74, 95% CI 1.50–5.00, respectively).

**TABLE 3:** Relative risk (RR) of a high anxiety level (STAI score ≥ 35), stratified by good or poor health (SF‐12 – baseline), by previous abnormal cervical cytology, by letter with information about screening result and by histology result of the LLETZ

	Before LLETZ	Immediately after LLETZ	1 month after LLETZ	6 months after LLETZ
	RR (95% CI)			
SF‐12^a^				
Good physical health	Ref.	Ref.	Ref.	Ref.
Poor physical health	1.81 (1.06–3.08)	0.82 (0.36–1.86)	1.64 (0.79–3.41)	2.74 (1.50–5.00)
Good mental health	Ref.	Ref.	Ref.	Ref.
Poor mental health	3.77 (2.12–6.70)	2.07 (1.03–4.17)	3.37 (1.59–7.15)	1.93 (1.06–3.51)
Previous abnormal cervical cytology (ASC‐US+)				
Normal cytology	Ref.	Ref.	Ref.	Ref.
Abnormal cytology	1.37 (0.90–2.10)	2.10 (1.13–9.92)	2.62 (1.43–4.79)	1.30 (0.74–2.78)
Letter with information about the screening result^b^				
Positive HPV (and normal cytology)	Ref.	Ref.	Ref.	Ref.
Abnormal cytology	1.1 (0.70–1.64)	0.68 (0.36–1.30)	1.31 (0.69–2.50)	0.90 (0.52–1.57)
Histology result^c^				
<CIN2	Ref.	Ref.	Ref.	Ref.
CIN2+	0.98 (0.62–1.56)	1.35 (0.71–2.58)	1.97 (1.07–3.63)	1.45 (0.84–2.51)

Abbreviations: RR, relative risk; STAI, State–Trait Anxiety Inventory.

^a^ Short Form 12 (SF‐12).

^b^ Information about the screening result was divided between a letter stating that they were HPV positive or a letter stating that they had abnormal cytology (ASC‐US+).

^c^ <CIN2, normal and CIN1; CIN2+, CIN2, CIN3, adenocarcinoma in situ and cancer. ASC‐US+ is defined as atypical squamous cells of undetermined significance (ASC‐US), low‐grade squamous intraepithelial lesion (LSIL), atypical squamous cells that cannot exclude high‐grade squamous intraepithelial lesion (ASC‐H) and high‐grade squamous intraepithelial lesion (HSIL).

We found no difference in risk of a high levels of anxiety from immediately after LLETZ, and one and six months after LLETZ in women who received a letter with the information of an abnormal cytology compared to women who received a letter with the information of a HPV‐positive test (RR 1.1, 95% CI 0.70–1.70; RR 0.68, 95% CI 0.36–1.30; RR 1.31, 95% CI 0.69–2.50; and RR 0.90, 95% CI 0.52–1.57, respectively) (Table 3). Compared with women diagnosed with <CIN2 from the LLETZ specimen, women diagnosed with CIN2+ could seem more likely to have high anxiety levels immediately after the LLETZ (RR 1.35, 95% CI 0.71–2.58), 1 month after the LLETZ (RR 1.97, 95% CI 1.07–3.63) and 6 months after the LLETZ (RR 1.45, 95% 0.84–2.51) (Table 3). However, not all findings were statistically significant.

Women with previous abnormal cervical cytology were more likely to have increased anxiety levels compared with women who had a normal screening history, especially immediately after colposcopy and LLETZ (RR 2.10, 95% CI 1.13–9.92) and at 1 month after LLETZ (RR 2.62, 95% CI 1.43–4.79) (Table 3).

We performed a sensitivity analysis changing the cut‐off value for defining a high level of anxiety (i.e. an STAI median score of ≥40), which did not change our main results (Table S2).

## 4 DISCUSSION

### 4.1 Main findings

This longitudinal study demonstrated that postmenopausal women with abnormal screening tests referred for diagnostic LLETZ at the first colposcopy visit, in general, had low anxiety levels. We observed increased anxiety levels in postmenopausal women prior to their colposcopy visit as compared with immediately after they were offered a diagnostic LLETZ. In most women the anxiety levels remained stable from immediately after the LLETZ to 1 and 6 months after the LLETZ. A subgroup of women with poor mental health were more likely to have increased anxiety levels before and up to 6 months after the LLETZ, as compared with women who had good mental health.

### 4.2 Strengths and limitations

To our knowledge, this is the first study to report on anxiety in postmenopausal women undergoing diagnostic LLETZ at their first colposcopy visit. A major strength in this study was that we used a validated Danish version of the STAI.^16^ We collected data from women prior to their colposcopy visit and up to 6 months afterwards to ensure that anxiety levels were measured after the women received the histopathologic results from their colposcopy visit. However, we did not use a stability anchor and had no control group. Therefore, we cannot exclude that the anxiety measured may be caused by other things affecting a woman’s life (e.g. illness, death in the near family, the Covid‐19 pandemic, etc.). It is difficult to know how the anxiety levels would have been in women undergoing colposcopy and biopsies only (i.e. without immediate LLETZ), as we did not include a control group. In this study, we used median values as the data were skewed. The median scores were lower than the mean scores and therefore our results are underestimates, as compared with other studies presenting mean scores (Q1, mean 33.4 vs median 32.0; Q2, mean 29.3 vs median 28.0; Q3, mean 31.1 vs median 30.0; and Q4, mean 31.6 vs median 29.0). Further, validation of the Danish STAI found the smallest detectable change to be 14.4 points, indicating that changes of less than 14.4 points may potentially be a result of measurement error.^16^ Even though several limitations in the validation study may have caused this high value, the differences found in this study are rather small and should be interpreted with caution.

We had a high response rate throughout the study (Q1, STAI ≥ 88.8% and SF‐12 ≥ 84.7), despite that women were asked to complete Q1 before being considered for eligibility. However, we cannot rule out potential selection bias as non‐responders could have had different anxiety levels compared with the women who responded. Finally, the small sample size makes the results less robust.

Our results may not be generalisable to younger women referred for colposcopy as both life circumstances and side effects are different for this group (e.g. risk of premature birth in younger women versus risk of stenosis in postmenopausal women).^25^ However, they may be generalisable to other settings offering diagnostic LLETZ to postmenopausal women.

### 4.3 Interpretation

Compared with other studies that measure anxiety levels before and after colposcopy, we found an overall low STAI median score for anxiety, despite that women in our study were informed that, if they were eligible, they would be offered a diagnostic LLETZ.^10^, ^11^, ^26^, ^27^, ^28^ This could partly be explained by differences in age distribution, as the median age in the present study was 67.7 years (IQR 61.6–70.3 years) compared with the above mentioned studies that had mean ages ranging from 29.0 to 45.6 years. Life circumstances (i.e. no longer responsible for minor children, illness, comorbidities, etc.) and the difference in side‐effects of treatment with increasing age (i.e. the risk of preterm birth is no longer relevant) could also contribute to the lower anxiety levels seen in the present study. However, studies on postmenopausal women and the psychological effect of referral for or diagnostic work‐up and treatment for abnormal cervical cancer screening test are limited. A recent review on the psycho‐social influences on older women (50–64 years) attending cervical screening concluded that the older women shared several barriers, such as fear of discomfort, pain and embarrassment, with their younger counterparts, and indicated that future studies on older women are needed.^29^ Contradictory to our results, Giannella et al. found that postmenopausal women had a greater negative psychological impact from LLETZ compared with premenopausal women.^30^ However, a meaningful comparison with this study is compromised as the previous study used a non‐standardised questionnaire and not the STAI, which could account for the observed differences.

Studies investigating the psychological impact of HPV screening has shown that a positive HPV result may lead to increased anxiety and concern about the possibility of developing cervical cancer.^31^, ^32^ Although not long lasting, women with low‐grade cytology who tested HPV positive had increased anxiety compared with women who had low‐grade cytology and who either tested HPV negative or did not have an HPV test performed.^32^, ^33^ These results are in line with a recent study by McBride et al. that investigated women undergoing HPV‐based screening at one of five primary HPV‐screening pilot sites in England.^34^ They found that, compared with a control group who had normal cytology, women screened for HPV with or without abnormal cytology had significantly higher anxiety levels.^34^ In the present study we did not find increased anxiety levels in women aged ≥45 years who were HPV positive as compared with women who had abnormal cytology. The underlying reason for this remains unclear. A Danish qualitative study focusing on women aged ≥60 years investigated the experiences of women with screening and colposcopy, including knowledge on and attitudes towards underdiagnosis/overtreatment, and their preferences for follow‐up. These women called for better information and more clear communication of the consequences of being HPV positive. Moreover, they had considerable intolerance towards clinical uncertainty and repeat testing and, despite the increased risk of overtreatment, they preferred a diagnostic LLETZ to gain closure.^7^

Similar to other studies,^26^, ^27^ we observed a decrease in anxiety levels from before to immediately after LLETZ. It is reasonable to believe that the immediate relief gained after thorough counselling by the physician and after the performance of the LLETZ leads to a decrease in anxiety levels. This is in line with the study by Sharp et al. that showed a similar decrease in anxiety from before colposcopy to 6 weeks after colposcopy or LLETZ using the Hospital Anxiety and Depression Scale.^35^ In the Danish validation study,^16^ we found that the smallest detectable change was 14 points. Despite some limitations in that validation study, an overall decrease from before to immediately after the LLETZ of 4 points (change in median STAI score from 32 to 28) in the present study, may not be clinically important and it is unlikely to cause a disturbance in the everyday life of the women. Nonetheless, a subgroup of women with a previous abnormal screening result had a two‐fold (RR 2.10) higher risk of anxiety immediately after the LLETZ, as compared with women who had a normal screening history. Moreover, a subgroup of women with poor mental health (SF‐12) had a nearly four‐fold (RR 3.77) higher risk of increased anxiety levels before LLETZ, as compared with women who had good mental health, and the anxiety level remained increased at 1 and 6 months after the LLETZ. Thus, even though these women’s anxiety levels may not only be affected by the intervention, we need to pay attention to the fact that there may be a vulnerable group of women who need special attention at the time of surgery and more individualised follow‐up and post‐treatment care.

## 5 CONCLUSION

Among women aged ≥45 years referred for colposcopy, diagnostic LLETZ was associated with a durable reduction in anxiety levels, which decreased from the baseline. Thus, the women generally seemed to experience an immediate relief of anxiety after the LLETZ, which remained stable for up to 6 months after the LLETZ. However, in a subgroup of women with poor mental health the anxiety levels were increased, suggesting that special attention should be given to this group of women.

## AUTHOR CONTRIBUTIONS

LWG, AH, BA, LKP, MBL and PB designed the study. LWG and MBL conducted the interpretation and analysis of the data, with feedback from the other authors. LWG drafted the article, with supervision, and PB, AH, BA, MBL and LKP provided critical revision of the article. All authors approved the final version for publication.

## FUNDING INFORMATION

Grants from the Health Research Foundation of Central Denmark Region (A3234), Dagmar Marshalls Foundation, Fabricant Einar Willumsens Mindelegat, Else and Mogens Wedell‐Wedellsborgs Foundation and the AP Moller Foundation (18‐L‐0124) funded this project. In addition LWG recieved a travel grant from Danish Cancer Society. None of the funders had an influence on the scientific process, including the decision to submit the article for publication.

## CONFLICT OF INTERESTS

LWG and AH have received speakers fees from Astra Zeneca outside the submitted work. LKP has received a speakers fee from MSD outside the submitted work. AH has received kits for p16 analysis from Roche Diagnostics, Denmark, outside the submitted work. BA has received HPV kits from Roche Diagnostic, outside the submitted work. LWG, PB and BA have received kits for p16/ki67 dual stain analysis from Roche Diagnostics, outside the submitted work. MBL has no conflicts of interest. Completed disclosure of interests form available to view online as supporting information.

## ETHICS APPROVAL

According to the EU General Data Protection Regulation (article 30), the project was listed under the record for processing activities for research projects in Central Denmark Region (ref. no. 1‐16‐02‐528‐18). The Central Denmark Region Committees on Health Research Ethics decided that according to the Danish Act on Research Ethics Review of Health Research Projects, this study should not be approved by the committees (ref. no. 1‐10‐72‐4‐17).

## Supporting information


Appendix S1

ICMJE

ICMJE

ICMJE

ICMJE

ICMJE

ICMJE

## Data Availability

The data set generated and analysed in this study is not available for the public, in accordance with Danish legislation. Data can be made available on request from researchers who meet the criteria for access to patient’s confidential data and upon approval from the Danish Data Protection Agency.
