# Supplementary material for: Levels of anxiety in women aged ≥45 years undergoing diagnostic large loop excision of the transformation zone: A longitudinal study
Source: BJOG. 2022 Oct 13;130(2):192–200. doi: 10.1111/1471-0528.17299 (PMC10091992; doi:10.1111/1471-0528.17299)
Supplement: Supplementary file 1 — Appendix S1 [file BJO-130-192-s004.docx]

**Supporting Table S1: Management of women after an abnormal screening result according to the Danish cervical cancer screening guidelines**

| **Result of primary screening method** | | **Reflex testing** | **Management** |
| --- | --- | --- | --- |
| **Women aged 30-59 who undergo cytology-based screening** | | | |
|  | ASC-US | Positive reflex HPV test | Direct referral to colposcopy |
|  |  | Negative reflex HPV test | Return to screening program |
|  | LSIL | Reflex HPV testing not recommended | Repeat cytology test after six months |
|  | High grade cytology^1^ | Reflex HPV testing not recommended | Direct referral to colposcopy |
| **Women aged 30-59 who undergo HPV-based screening** | | | |
|  | HPV positive | Triage with low grade^2^ and additional triage positive^3^ | Direct referral to colposcopy |
|  |  | Triage with low grade^2^ and additional triage negative^3^ | Repeat HPV testing after one year |
|  |  | Triage with high grade^1^ cytology | Direct referral to colposcopy |
|  |  | Normal cytology on cytology triage | Repeat HPV testing after one year |
| **Women aged 60-64 who undergo HPV-based screening** | | | |
|  | Positive for HPV 16 or 18^4^ | Not required | Direct referral to colposcopy |
|  | Positive for other HPV types | ASC-US or worse on reflex cytology | Direct referral to colposcopy |
|  |  | Normal on reflex cytology | Repeat HPV test after one year^5^ |

Abbreviations: HPV: human papilloma virus. ^1^ High grade cytology: HSIL: High-grade squamous intraepithelial lesion, Atypical squamous cells-cannot exclude HSIL (ASC-H), Atypical glandular cells (AGC), Adenocarcinoma in situ (AIS). ^2^ Low grade: ASC-US: atypical squamous cells of undetermined significance and LSIL: low-grade squamous intraepithelial lesion. ^3^ Additional triage from January 2021 (type of triage dependent on region). In Central Denmark Region additionally triage is p16/Ki67 dual stain. ^4^ Including high risk HPV other types in combination with 16 and/ or 18. ^5^ Two positive tests with persistent hrHPV other types will lead to referral to colposcopy from January 2021. Before January 2021 women were referred using ≥ASC-US as threshold.

**Table S2: Relative risk (RR) of a high anxiety level (STAI score ≥40) stratified by good and poor health (SF-12 - baseline), previous abnormal cervical cytology, information about screening, and histology result of the LLETZ**

|  | **Before LLETZ** | **Immediately after LLETZ** | **One month after LLETZ** | **Six months after LLETZ** |
| --- | --- | --- | --- | --- |
|  | **RR (95% CI)** | | | |
| **SF-12^1^** | | | | |
| *Good physical health* | Ref | Ref | Ref | Ref |
| *Poor physical health* | 2.19  (1.04 - 4.61) | 1.32  (0.34 – 5.09) | 1.92  (0.78 – 4.73) | 3.84  (1.84 – 8.00) |
| *Good mental health* | Ref | Ref | Ref | Ref |
| *Poor mental health* | 6.22  (2.52 – 15.33) | 2.07  (0.56 – 7.67) | 3.11  (1.23 – 7.85) | 2.07  (1.03 – 4.17) |
| **Previous abnormal cervical cytology (ASC-US+)** | | | | |
| *Normal cytology* | Ref | Ref | Ref | Ref |
| *Abnormal cytology* | 1.67  (0.97 – 2.87) | 2.27  (0.71 – 7.25) | 2.52  (1.14 – 5.56) | 1.42  (0.73 – 2.75) |
| **Information received about the screening result^2^** | | | | |
| *Positive HPV (and normal cytology)* | Ref | Ref | Ref | Ref |
| *Abnormal cytology* | 0.90  (0.52 – 1.57) | 1.27  (0.38 – 4.23) | 1.46  (0.63 – 3.38) | 0.99  (0.51 – 1.92) |
| **Histology result^3^** | | | | |
| *<CIN2* | Ref | Ref | Ref | Ref |
| *CIN2+* | 0.74  (0.39 – 1.40) | 0.84  (0.23 – 3.05) | 2.19  (0.99 – 4.86) | 1.44  (0.75 – 2.78) |

## Abbreviations: RR: Relative risk. STAI: State Trait Anxiety Inventory. ^1^: Short Form (SF)-12, SF-12 was not a part of Q2. ^2^: Information about the screening result was divided into women who received a letter with the result of the screening test either stating that they were HPV positive or had an abnormal cytology (ASC-US+). ^3^: <CIN2: Normal and CIN1, CIN2+: CIN2, CIN3, AIS, and cancer. ASC-US+ is defined as: ASC-US, LSIL, ASC-H and HSIL

**Figure S1. Timeline for the questionnaires**

Six months after LLETZ

One month after LLETZ

Immediately after LLETZ

Questionnaire 1

STAI

SF-12

Questionnaire 2

STAI

Questionnaire 3

STAI

SF-12

Questionnaire 4

STAI

SF-12

Before LLETZ

Abbreviation: LLETZ: Large loop excision of the transformation zone. STAI: state trait anxiety inventory. SF-12: Short form 12
